# Nickel Cobaltite Functionalized Silver Doped Carbon Xerogels as Efficient Electrode Materials for High Performance Symmetric Supercapacitor

**DOI:** 10.3390/ma13214906

**Published:** 2020-10-31

**Authors:** Madlin A. Wasfey, Abdalla Abdelwahab, Francisco Carrasco-Marín, Agustín F. Pérez-Cadenas, H. H Abdullah, I. S. Yahia, Ahmed Ali Farghali

**Affiliations:** 1 Nanotechnology Lab, Electronics Research Institute, El Nozha, Cairo 11311, Egypt; madlen.wasfy@yahoo.com (M.A.W.); Haythem@eri.sci.eg (H.H.A.); 2 Materials Science and Nanotechnology Department, Faculty of Postgraduate Studies for Advanced Sciences, Beni-Suef University, Beni-Suef 62511, Egypt; ahmedfarghali74@yahoo.com; 3 Carbon Materials Research Group, Department of Inorganic Chemistry, Faculty of Sciences, University of Granada, Campus Fuentenueva s/n, 18071 Granada, Spain; fmarin@ugr.es; 4 Unit of Excellence in Chemistry applied to Biomedicine and the Environment of the University of Granada, 18071 Granada, Spain; 5 Research Center for Advanced Materials Science (RCAMS), King Khalid University, Abha 9004, Saudi Arabia; isyahia@gmail.com; 6 Advanced Functional Materials & Optoelectronic Laboratory (AFMOL), Department of Physics, Faculty of Science, King Khalid University, Abha 9004, Saudi Arabia; 7 Nanoscience Laboratory for Environmental and Bio-medical Applications (NLEBA), Semiconductor Lab., Physics Department, Faculty of Education, Ain Shams University, Roxy, Cairo 11757, Egypt

**Keywords:** supercapacitors, carbon xerogel, silver nanoparticles, nickel cobaltite, hybrid electrode materials, porous structure

## Abstract

Introducing new inexpensive materials for supercapacitors application with high energy density and stability, is the current research challenge. In this work, Silver doped carbon xerogels have been synthesized via a simple sol-gel method. The silver doped carbon xerogels are further surface functionalized with different loadings of nickel cobaltite (1 wt.%, 5 wt.%, and 10 wt.%) using a facile impregnation process. The morphology and textural properties of the obtained composites are characterized by X-ray diffraction (XRD), scanning electron microscopy (SEM), transmission electron microscopy (TEM), and nitrogen physisorption analysis. The silver doped carbon xerogels display a higher surface area and larger mesopore volume compared to the un-doped carbon xerogels and hierarchically porous structure is obtained for all materials. The hybrid composites have been utilized as electrode materials for symmetric supercapacitors in 6 M KOH electrolyte. Among all the hybrid composites, silver doped carbon xerogel functionalized with 1 wt.% nickel cobaltite (NiCo1/Ag-CX) shows the best supercapacitor performance: high specific capacitance (368 F g^−1^ at 0.1 A g^−1^), low equivalent series resistance (1.9 Ω), high rate capability (99% capacitance retention after 2000 cycles at 1 A g^−1^), and high energy and power densities (50 Wh/Kg, 200 W/Kg at 0.1 A g^−1^). It is found that the specific capacitance does not only depend on surface area, but also on others factors such as particle size, uniform particle distribution, micro-mesoporous structure, which contribute to abundant active sites and fast charge, and ion transfer rates between the electrolyte and the active sites.

## 1. Introduction

The energy crisis is one of the main problems facing our world today, especially energy production and storage [1,2]. Uses of fossil fuels as the source of energy production is certainly having a severe impact on ecology due to the production of greenhouse gases like carbon dioxide CO_2_, in addition, the discovery of the fossil fuel has been decreasing since 1969 [3]. In addition, many developed countries are on their way to substitute fossil fuel and natural gas with biogas for economically and environmentally reasons [4]. It should be also pointed out that biowaste pyrolysis is one of the most profitable methods of carbon sequestration [5]. So, we have to search for alternative sources for energy production and how to store this energy to be able to use it during peak demand. Lithium ion battery (LIB) and supercapacitors (SCs) are the two main devices for energy storage [6]. SCs are considered the bridge between LIB (high energy density) and conventional capacitors (high power density). The supercapacitors global market size is increasing every year and it is expected to reach about $16.95 billion by 2027. SCs possess a long life time, high power density, and much shorter charging/discharging times. On the other hand, SCs face the main problem of lower energy density between 5–20 Wh/kg comparable with (LIB) [6,7]. A supercapacitor is a device suitable for the rapid storage and release of energy; it consists of two porous electrodes immersed in electrolyte, i.e., organic, aqueous, or ionic electrolyte, and separated by a porous membrane that allows the migration of electrolyte ions [8,9]. A supercapacitor can be used in many applications like consumer electronics, memory back-up systems, and low-emission hybrid electric vehicles [10]. Based on their mode of energy storage, supercapacitors can be classified into two types: (i) Electrochemical double layer capacitors (EDLCs), where energy storage occurs through electrostatic storage which takes place by separating of charges in a Helmholtz double layer at the interface between the electrode and electrolyte interface, so the capacitance depends on the surface area of the used electrode. (ii) Pseudocapacitors, where energy storage occurs through Faradaic electrochemical storage with electron charge transfer by redox reactions [11].

Carbon materials such as activated carbon (AC) [12], graphene [13], reduced graphene oxide, carbon nanotubes (CNTs), and carbon gel [14] are widely used in EDLCs due to unique physiochemical properties such as high specific surface area, thermal stability, chemical stability, and conductivity [15,16]. However, the capacitance of carbon-based supercapacitors is still limited, for example, activated carbon nanofibers have capacitance of 60 F·g^−1^ in 2 M sulfuric acid (H_2_SO_4_) electrolyte [17]. Modification of multiwalled carbon nanotubes (MWNTs) with chemical activation by potassium hydroxide (KOH) increases the specific capacitance of nanotubular material from 15 to 90 F·g^−1^ [18,19]. Although graphene and its composites materials have high specific capacitances compared to others carbon-based materials, its lamellar structure causes leaches for the dopant, especially in the liquid electrolyte.

Recently, carbon xerogel (CX) was used as an electrode material for SCs due to features such as its open three-dimensional (3D) structure network which prevent leaches of the dopant during its application in the liquid phase. In addition, it has high specific surface area, good electrical conductivity, and control of porosity during its synthesis [20]. The special porous structure of carbon xerogels can give productive dispersion/mass exchange and an enormous number of active sites for the electrostatic attraction and movement of ions/molecules and permits steady and continued transport of electrolyte ions and electrons to the surface, which prompts amazing electrochemical performance for supercapacitor [14,20]. Moreover, carbon gels can be doped with several transition metals in order to gain the advantages of both. Z. Zapata-Benabithe et al. [21] used carbon aerogels doped with copper and silver electrodes for supercapacitors applications. The obtained gravimetric capacitances were 192 and 100 F·g^−1^ for copper and silver doped carbon aerogels, respectively. X. Liu et al. [22] prepared nitrogen doped carbon xerogel (NCXs) for supercapacitors, the surface area of the prepared material was 4279 m^2^ g^−1^, which delivers a specific capacitance of 271 F g^−1^.

Transition metal oxides such as (RuO_3,_ NiO, Co_3_O_4_, and MnO_2_) have high specific capacitance and are widely used for pseudocapacitors which store charges by faradic reaction. Among these, RuO_2_ has been broadly examined as a promising applicant due to its better conductivity and high specific capacitance. However, its enormous scale application is constrained by the high cost and rarity of the Ru element [23,24]. In contrast, binary metal oxide such as (NiCo_2_O_4,_ MnCo_2_O_4_, and ZnCo_2_O_4_) have low cost, abundant resource, and environmental friendliness. Especially, NiCo_2_O_4_ possesses better electrical conductivity and richer active sites than mono-metal oxides (NiO, Co_3_O_4_). Therefore, NiCo_2_O_4_ is a promising electrode material for energy storage devices including fuel cells, lithium-ion batteries (LIBs), and electrochemical capacitors (ECs) [25,26,27,28]. R.B. Marichi et al. prepared 3D oxygen-rich carbon aerogel/NiCo_2_O_4_ which has a specific capacitance of 160 F·g^−1^ at 0.5 A/g for two electrode configuration and 473 F g^−1^ at 5 mV·s^−1^ [29]. Stability is another important factor in supercapacitors application, and unfortunately, metal oxides suffer from poor cycling stability. M. Eskandari et al. prepared NiCo_2_O_4_/MWCNT and NiCo_2_O_4_/PANI, the stability was 88% and 70% after a 2000 cycle, respectively [25].

The hydrothermal method is a well-known process for preparation of metal oxides with different morphologies, but unfortunately, this method destroys the surface area of carbon which is an important parameter for efficient supercapacitor. M.M. EL-Deeb et al. prepared carbon xerogel doped with NiCo_2_O_4_ for methanol electro-oxidation, he found that the surface area S_BET_ of carbon xerogel after the hydrothermal process drops from 701.9 to 64.0 m^2^ g^−1^ [30].

NiCo_2_O_4_/silver doped carbon xerogel composite has not been studied before as supercapacitors electrodes. A published work [31] investigated the carbon xerogel with manganese oxide as supercapacitors electrodes in a symmetric and asymmetric configuration. In their study, they found a relation between texture properties of carbon and pore size distribution with the obtained capacitance, and the highest obtained capacitance was 213 F·g^−1^ for asymmetric configuration.

In this work, silver doped carbon xerogel (Ag-CX) was prepared by the sol gel method from resorcinol and formaldehyde monomers. Silver doped carbon xerogel is further functionalized with NiCo_2_O_4,_ (NiCo_2_O_4_/Ag-CX) by the impregnation method to keep the surface area and porous structure of carbon xerogel. Different mass ratios of NiCo_2_O_4_, namely 1 wt.%, 5 wt.%, and 10 wt.%, were loaded into the carbon xerogel matrix. Symmetric hybrid SCs with hybrid active materials were fabricated and tested as supercapacitors electrodes and the impact of different ratios of NiCo_2_O_4_ on carbon xerogel and electrochemical performance was studied and discussed in 6 M KOH as an aqueous electrolyte.

## 2. Materials and Methods

### 2.1. Materials

All the purchased chemicals, including resorcinol, formaldehyde, silver acetate, cobalt acetate (Co(Ac)_2_·6H_2_O), and nickel acetate (Ni(Ac)_2_·6H_2_O) are of analytical grade. The chemicals were used without any further treatment.

### 2.2. Preparation of Porous Carbon Xerogel (CX)

The porous CX was prepared by dissolving a mixture of resorcinol (R) and formaldehyde (F) (R:F = 1:2 molar ratio) in water (W) (R:W = 1:17 molar ratio) using sodium carbonate (Na_2_CO_4_) as a catalyst (C) (R:C = 300:1 molar ratio). These were mixed using a magnetic stirrer to get a homogeneous solution that was poured into glass molds (45 cm length × 0.5 cm diameter). The glass molds were kept for one day at 40 °C followed by five days at 80 °C. The resulting hydrogel rods were cut and stored in acetone for three days to exchange the solvent media so that the surface area and porosity are maintained during the drying process. The organic gels were thereafter dried using microwave-assisted heating. The dried organic gels were carbonized at 900 °C (the ramp rate was set at 1 °C/min) for 2 h under N_2_ stream in a tube furnace and were then ground into CX powder.

### 2.3. Preparation of Silver Doped Carbon Xerogel (Ag-CX)

Silver doped carbon xerogel (Ag-CX) was prepared following the same procedure of preparing CX, except that silver acetate (CH₃CO₂Ag) was used as the polymerization catalyst, instead of Na_2_CO_4_. The amount of elemental silver in the carbon matrix was estimated to be 1 wt.% of the final carbon weight.

### 2.4. NiCo_2_O_4_ Functionalization of Ag-CX (NiCo_2_O_4_/Ag-CX)

The dried silver doped carbon organic gel was used as the substrate for the growth of NiCo_2_O_4_. Salts of Ni(Ac)_2_·6H_2_O (0.036 gm, 0.5 mmol) and Co(Ac)_2_·4H_2_O (0.07 gm, 1 mmol) were dissolved in 5 mL deionized water and then ultrasonicated for 15 min. The solution was poured dropwise into the silver doped carbon organic gel, which was then dried using microwave-assisted heating. The precursor was then carbonized as discussed before in Section 2.2. Three different loadings of NiCo_2_O_4_ (1 wt.%, 5 wt.%, and 10 wt.%) grafted on the Ag-CX matrix, were prepared and labeled as (NiCo1/Ag-CX), (NiCo5/Ag-CX), and (NiCo10/Ag-CX), respectively.

### 2.5. Structural Characterization

The electrode samples were characterized by different experimental techniques. X-ray diffractometer (XRD) (BRUKER, Rivas-Vaciamadrid, Spain) (PANalytical Empyrean diffractometer, CuKα radiation, wavelength λ = 1.54045Å, accelerating voltage 40 KV, current 30 mA, scan range 5–80°, and scan step 0:05°) was employed to identify the phase structures of the composites. The surface morphology of the samples was characterized using scanning electron microscopy (SEM, Model Quanta 250 FEG) (ThermoFisher, Hillsboro, OR, 97124 USA) and transmission electron microscope (TEM, JOEL JEM-2100) (Joel, Akishima, Tokyo, JAPAN). Surface area and porosity analysis were performed by N2 adsorption/desorption at 77 K using surface area analyzer (Quadrasorb, Quantachrome instrument, Quadrasorb, Boynton Beach, FL, USA). Before the porosity analysis, the samples were outgassed for 12 h at 110 °C under high vacuum conditions (10−6 mbar). The specific surface area was determined by applying the Brunauer-Emmett-Teller (BET) equation to the recorded N2 isotherms. The porosity of the samples was evaluated by applying the Barret–Johner–Halenda (BJH) method to the adsorption part of the isotherms.

### 2.6. Preparation of Symmetric Hybrid Device and Electrochemical Measurement

The electrochemical electrodes were prepared by mixing the pristine/modified carbon xerogels (90 wt.%) with polytetrafluoroethylene (PTFE) (60% dispersion in water). The mixture was pasted onto disk-shaped graphitic substrates (0.9 cm in diameter). Afterwards, the electrodes were mechanically pressed with 2 bar pressure and left to dry overnight at 100 °C. Prior to the electrochemical characterizations, the electrodes were soaked in the respective electrolyte for 48 h. Symmetric supercapacitors were built between two electrode disks of the same weight in a Perfluoroalkoxy alkanes (PFA) Swagelok^®^ cell. The electrochemical behavior of the supercapacitors was investigated by performing cyclic voltammetry (CV), galvanostatic charge–discharge, and electrochemical impedance spectroscopy (EIS) measurements in 6 M potassium hydroxide (KOH) electrolyte at 25 °C using a two-electrode system with a potentiostat (EC-lab VMP system, Biologic, Science Instruments) (BioLogic, Seyssinet-Pariset, France). The cyclic voltammetry and galvanostatic charge-discharge tests were carried out at different scan rates (3, 5, 10, 20, and 50 mV·s^−1^) and different currents densities (0.1, 0.5, 1, 3, and 5 A·g^−1^), respectively.

## 3. Results and Discussions

### 3.1. Physicochemical Characteristics

**Figure 1:**
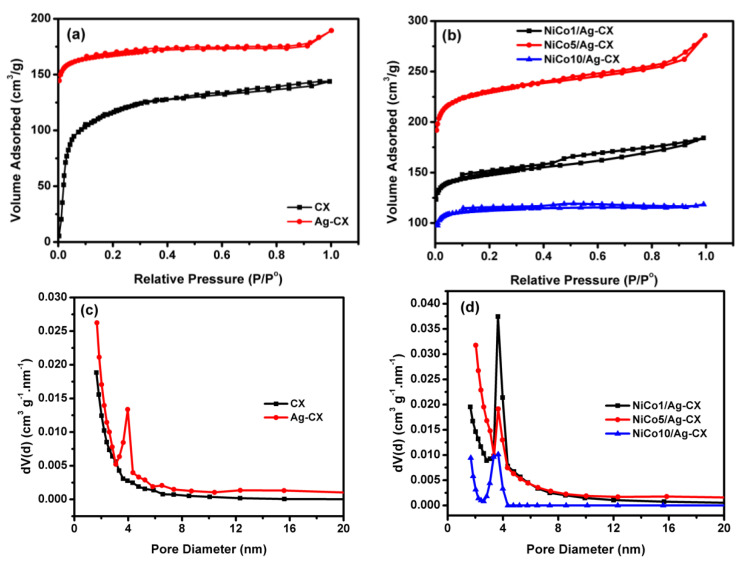
N_2_ adsorption–desorption isotherms of (**a**) CX and Ag-CX, (**b**) NiCo1/Ag-CX, NiCo5/Ag-CX, and NiCo10/Ag-CX. BJH Pore size distributions for (**c**) CX and Ag-CX, (**d**) NiCo1/Ag-CX, NiCo5/Ag-CX, and NiCo10/Ag-CX.

Figure 1 depicts the N_2_ adsorption/desorption isotherms of the different electrode materials at 77 K together with their particle size distributions. Figure 1a shows the isotherms for blank carbon xerogel (CX) and silver doped carbon xerogel (Ag-CX), which are closely resemble type I, which is characteristic for microporous materials [21,32]. The sharp increase in nitrogen gas uptake for CX at low relative pressures (P/P° < 0.2) due to micropore filling, indicates that most of carbon xerogel pores are in the micropore range. It also seems that incorporation of Ag in the carbon matrix as a polymerization catalyst increases the carbon yield which appeared in higher micropore volume (W_o_) compared to pristine CX, Table 1. The isotherms for the NiCo_2_O_4_ functionalized silver doped carbon xerogels with three different loadings of nickel cobaltite are shown in Figure 1b. The isotherms for NiCo_2_O_4_/Ag-CX, Figure 1b, are a combination between type I and type IV isotherms. The hysteresis loops are detected for all NiCo_2_O_4_ functionalized samples. However, different hysteresis loops are observed for NiCo_2_O_4_ functionalized samples, for example, the hysteresis loops of NiCo5/Ag-CX and NiCo10/Ag-CX are of type H4 [32,33], while the hysteresis loop for NiCo1/Ag-CX is mainly of type H2, which is found for micro-mesoporous carbons. These differences in pore geometries clarify that the silver doped carbon xerogel functionalized with 1 wt.% of nickel cobaltite (NiCo1/Ag-CX) has the highest mesopore volume, which would enable a facile transport of electrolyte ions into the active sites of the carbon matrix. In addition, all materials have additional large macropores, as appeared with some condensation steps at high relative pressures (P/P° > 0.9). Due to the presence of all three types of pores, the materials are described as hierarchically porous carbon materials.

The BJH Pore size distributions for all samples are represented in Figure 1c,d. The development of the mesopore volume, with silver doping is also confirmed with a sharp peak for Ag-CX, Figure 1c, in the range between 3.1 to 4.5 nm and the maximum pore size is about 3.9 nm. In addition, all NiCo_2_O_4_ functionalized samples have development in the mesopore volume, Figure 1d. However, the volume of nitrogen gas adsorbed onto NiCo1/Ag-CX is the highest when compared to NiCo5/Ag-CX or NiCo10/Ag-CX. These observations show that not only the surface area is important for carbon-based supercapacitors, but also the development in the mesopores volumes. Table 1 summarizes the data obtained from the analysis of the isotherms using the BET equation in a relative pressure range between 0.02 to 0.09.

**Figure 2:**
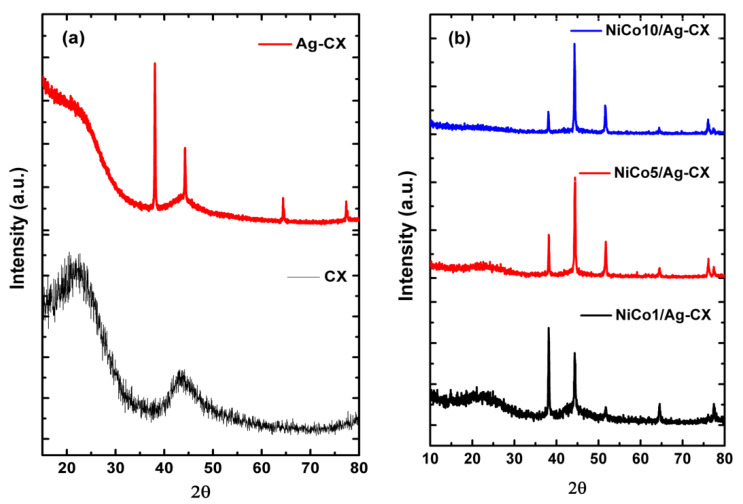
XRD patterns of (**a**) CX and Ag-CX, (**b**) NiCo1/Ag-CX, NiCo5/Ag-CX, and NiCo10/Ag-CX.

Figure 2 shows the XRD patterns of the as-prepared materials. As shown in Figure 2a, CX displays two peaks centered at 2θ of about 23.3° and 43.3°, which correspond to the (002) and (101) planes of graphite, respectively. The broadness of these two peaks indicates that CX possesses an amorphous structure. The silver doped carbon xerogel pattern shows a more graphitic carbon structure with diffraction peaks centered at 38.1°, 44.3°, 64.4°, and 77.5°, which can be indexed to the (111), (200), (220), and (311) planes of fcc-Ag, respectively [34].

Figure 2b represents the XRD patterns of the nickel cobaltite functionalized silver doped carbon xerogels. The XRD patterns reveal that the nickel and cobalt ions are completely reduced to fcc-metallic states after pyrolysis at 900 °C [28,30,35]. We propose that nickel and cobalt ions incorporated into the gel framework underwent progressive reduction to form metal carbide at moderate pyrolysis temperatures and elemental metals and metal oxides at high pyrolysis temperatures [36]. The functionalized samples show a diffraction peak at 51.8°, which can be assigned to the (200) plane of NiCo_2_O_4_ [37]. The intensity of the graphitic (002) peak as well as the sample conductivity decreases with increasing NiCo_2_O_4_ loadings, which means a decrease in the sample conductivity in the order from NiCo1/Ag-CX to NiCo5/Ag-CX and NiCo10/Ag-CX [14]. The Scherrer equation was applied to the XRD patterns in order to estimate the mean particle size (d_XRD_); the results are summarized in Table 2. NiCo1/Ag-CX has a mean particle size of about 26.7 nm which is the smallest among all samples. It is worth mentioning that a decrease in charge transfer resistance, R_CT_, is obtained as the catalyst nanoparticles become smaller [38].

**Figure 3:**
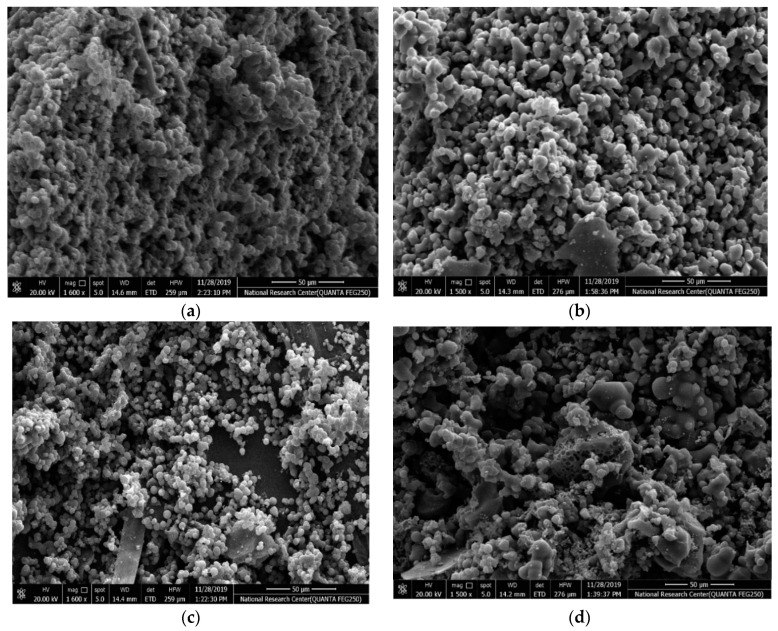
SEM images of samples (**a**) CX, (**b**) Ag-CX, (**c**) NiCo1/Ag-CX, and (**d**) NiCo5/Ag-CX.

The surface morphology of the different samples is characterized by SEM as shown in Figure 3a–d. The CX framework (Figure 3a) manifests a 3D porous structure that consists of spherical interconnected particles, which is in agreement with the reported morphology of resorcinol-formaldehyde carbon gel [39]. Such network structures are beneficial for achieving both high electrical conductivity via the connected monolithic walls and high ionic mobility and surface accessibility via the porous channels [40]. It is found that the CX monolith maintains its interconnected network structure. The Ag-CX (Figure 3b) and NiCo_2_O_4_ functionalized Ag-CX (Figure 3c–d) samples exhibit a more open structure compared to pristine CX.

**Figure 4:**
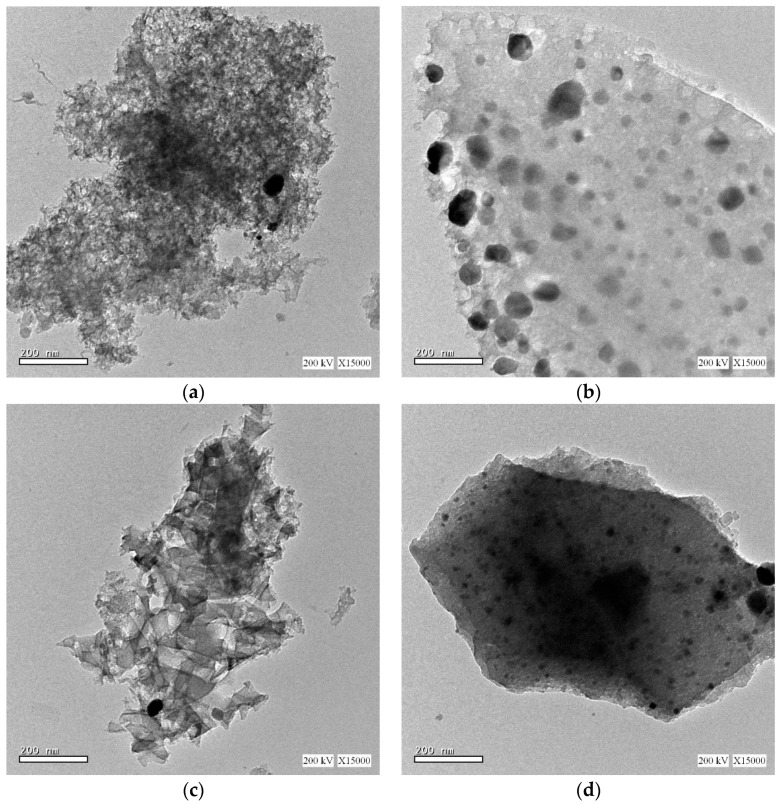
TEM images of (**a**) Ag-CX, (**b**) NiCo1/Ag-CX, (**c**) NiCo5/Ag-CX, and (**d**) NiCo10/Ag-CX.

The interior surface morphologies of the samples are investigated using high resolution transmission electron microscopy (HRTEM), as represented in Figure 4. The HRTEM images reveals that the Ag and Ag/NiCo2O4 catalytic nanoparticles are uniformly dispersed in the carbon xerogel matrix, as illustrated in Figure 4a–d, respectively. Moreover, the catalytic nanoparticles are observed to grow with a narrow particle size distribution until an average particle size, comparable to the size estimated by XRD, is reached.

### 3.2. Electrochemical Study

**Figure 5:**
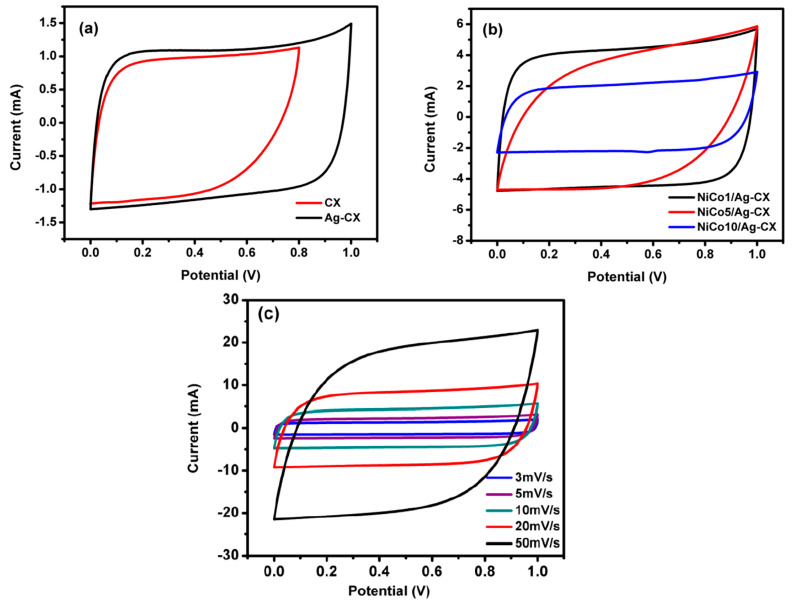
CV curves for (**a**) CX and Ag-CX at 5 mV·s^−1^, (**b**) NiCo1/Ag-CX, NiCo5/Ag-CX, and NiCo10/Ag-CX at 10 mV·s^−1^, and (**c**) NiCo1/Ag-CX at different scan rates.

The electrochemical measurements were carried out following standardized test methods [41]. The cyclic voltammetry (CV) tests were conducted in a 6 M KOH aqueous electrolyte at different scan rates and within a voltage window of 0–1V for all samples except CX, which was measured within a potential window of 0–0.8 V. As shown in Figure 5a–c, the supercapacitors exhibit a near-rectangular CV curves, which indicate that they possess a close-to-ideal double-layer capacitive behavior. The cell capacitance (*C*) at various scan rates was calculated using Equation (1), whereas the specific capacitance (*C_S_*) was calculated using Equation (2) [41,42]:

(1) C = ∫Idv/vΔv,

(2) Cs = 4C/m,

where ∫Idv is the integrated area under the CV curve, v is the scan rate, Δv is the voltage window, and m is the mass of the active material of the two electrodes. The factor 4 was used to calculate capacitance for a three electrodes equivalent system in order to make better comparison with the literature.

The estimated specific capacitances for CX, Ag-CX, NiCo1/Ag-CX, NiCo5/Ag-CX, and NiCo10/Ag-CX are listed in Table 3. It is obvious that the specific capacitance increases as the scan rate decreases. This behavior can be attributed to the fact that low scan rates allow enough time for the electrolytic ions to diffuse into the inner and less accessible micropores of the carbon xerogels. The introduction of Ag nanoparticles to the carbon xerogel matrix enhances its conductivity and improves the capacitance from 100 to 172 F·g^−1^ at a scan rate of 5 mV s^−1^ while maintaining a quasi-rectangular shape (Figure 5a). The improved specific capacitance of the Ag-CX sample may be attributed to the fact that silver nanoparticles participate in faradaic reversible redox reactions that lead to a pseudocapacitance contribution [38].

The capacitance of Ag-CX is further improved when it is functionalized with NiCo_2_O_4_, as indicated by the higher current densities associated with the functionalized electrodes (Figure 5b). The highest capacitance density and the best reversibility is registered for the NiCo1/Ag-CX supercapacitor, as illustrated in Figure 5b,c. The excellent performance of the NiCo1/Ag-CX electrode material is ascribed to the uniform distribution of the smallest Ag/NiCo1 nanoparticles in the well-developed mesopore CX matrix.

The charge/discharge mechanism for NiCo/Ag-CX may involve: (a) ionic transport through the electrolyte, (b) adsorption/desorption of ions at the surface active sites of the electrode, and (c) ions insertion and extrication into/from bulk, according to the following redox reactions [23,43]:

(3) $$\left({\left({\mathrm{NiCo}}_{2}O_{4}\right)}_{\mathrm{surface}}\;+{\;\mathrm{OH}}^{-}\;+{\;H}_{2}O\;↔\;\mathrm{NiOOH}\;+\;\left(2\mathrm{CoOOH}\right){\;}_{\mathrm{surface}}\;+\;2e^{-}\right)$$

(4) $$\left(\left(\mathrm{CoOOH}\right){\;}_{\mathrm{surface}}\;+{\;\mathrm{OH}}^{-}\;↔{\;\mathrm{CoO}}_{2}\;+{\;H}_{2}O\;+e^{-}\right)$$

(5) $$\left({\left({\mathrm{NiCo}}_{2}O_{4}\right)}_{\mathrm{bulk}}\;+{\;\mathrm{OH}}^{-}+{\;H}_{2}O↔\;\mathrm{NiOOH}\;+\left(2\mathrm{CoOOH}\right){\;}_{\mathrm{bulk}}\;+\;2e^{-}\right)$$

(6) $$\left(\left(\mathrm{CoOOH}\right){\;}_{\mathrm{bulk}}\;+{\;\mathrm{OH}}^{-}\;↔{\;\mathrm{CoO}}_{2}\;+{\;H}_{2}O\;+e^{-}\right)$$

This denotes that not only the surface area is important for EDLCs, but also the particle size distribution and the degree of mesoporosity. This may explain the lower supercapacitive performance of NiCo5/Ag-CX and NiCo10/Ag-CX when compared with NiCo1/Ag-CX, taking into account that high NiCo_2_O_4_ concentrations may cause permeability reduction and pore-blocking effects [44,45].

Galvanostatic charge/discharge tests were performed in 6M KOH within a voltage window of 0–1 V at different current densities. The gravimetric specific capacitance Cs was estimated using Equation (7) [41,42]:

(7) $$C_{s}=4Idt/m\Delta v$$

where *I* is the applied current, dt is the discharging time, m is the total mass (in grams) of the electrode active material, Δv is the operating voltage window. Here again, the factor 4 was used to calculate capacitance for a three electrodes equivalent system.

**Figure 6:**
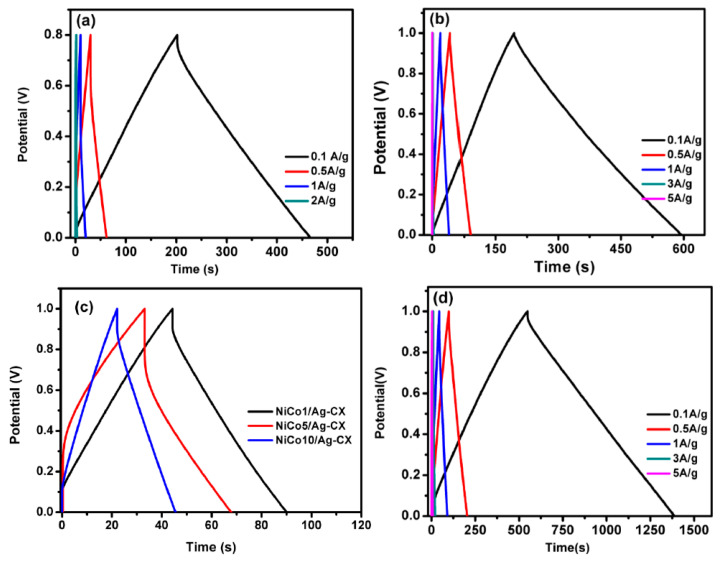
Charge/discharge curves for (**a**) CX at different current densities, (**b**) Ag-CX at different current densities, (**c**) NiCo1/Ag-CX, NiCo5/Ag-CX, and NiCo10/Ag-CX at 1 A g^−1^, and (**d**) NiCo1/Ag-CX at different current densities.

The charge/discharge curves for all samples at different current densities are shown in Figure 6a–d. The electrodes display a nearly triangular shape with good symmetry at various current densities, implying high columbic efficiency [27,46,47]. The functionalized electrodes exhibit excellent capacitive properties and superior reversible redox reaction, as reflected in their greater charge/discharge time. The NiCo1/Ag-CX electrode shows an ultrahigh specific capacitance of 368 F·g^−1^ at 0.1A g^−1^ (Table 3). A decrease in specific capacitance was observed for NiCo5/Ag-CX and NiCo10/Ag-CX as compared to NiCo1/Ag-CX, which may be due to their lower surface areas and mesopore volumes. This indicates that NiCo1/Ag-CX is the choice candidate for highly efficient supercapacitor applications, in which the kinetics of ion and electron transport is promoted at the electrode/electrolyte interface. The galvanostatic charge/discharge performance of the NiCo1/Ag-CX electrode at different current densities is shown in Figure 6a. It is clear that the charge/discharge time decreases as the current density increases. This is mainly because of the shorter time available for the electrolyte ions to diffuse into the interior surfaces of the electrode. Interestingly, the NiCo1/Ag-CX electrode maintains high specific capacitances even at high current densities, which demonstrates good performance in terms of stability and fast responses.

**Figure 7:**
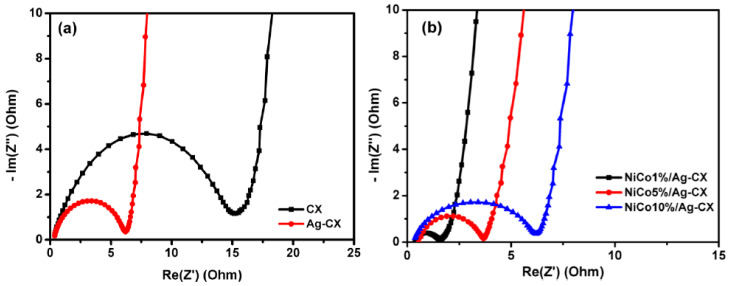
Nyquist plot for (**a**) CX and Ag-CX and (**b**) NiCo1/Ag-CX, NiCo5/Ag-CX, and NiCo10/Ag-CX.

Electrochemical impedance spectroscopy (EIS) has been widely utilized in evaluating the electrochemical performance of supercapacitors. We carried out EIS tests on the different CX electrodes by applying a frequency range from 100 KHz to 1 mHz with a sinusoidal signal amplitude of 10 mV. Figure 7 displays the Nyquist plots for CX, Ag-CX, NiCo1/Ag-CX, NiCo5/Ag-CX, and NiCo10/Ag-CX. The imaginary part of the Nyquist plots sharply increases and a nearly vertical line is observed in the low frequency range owing to the dominant double-layer capacitive behavior of the electrodes. Furthermore, all the electrodes exhibit a semicircle in the high frequency range due to the charge/transfer resistance (R_CT_) at the electrode/electrolyte interface. The larger the diameter of the semicircle, the higher the resistance [48]. The intersection of the semicircle with the real axis at around −45° represents the equivalent series resistance (ESR). The extracted ESR values of the different electrodes are listed in Table 3. We observed that the R_CT_ and the ESR decrease in the following order: CX → Ag-CX → NiCo10/Ag-CX → NiCo5/Ag-CX → NiCo1/Ag-CX. The low R_CT_ and ESR values of the NiCo1/Ag-CX supercapacitor further confirms high charge transfer and ion diffusion rates between the electrolyte and the active electrode material, in good agreement with the cyclic voltammetry and galvanostatic charge/discharge results.

The energy and power densities for two-electrode SC configuration can be estimated from the following Equations [26]:

(8) $$E\;\left(Wh/Kg\right)\;=\;Cs\;\;\left(F/g\right)\;\Delta V^{2}\;\left(v\right)÷7.2$$

(9) $$P\;\left(W/Kg\right)\;=\;\frac{E\;(Wh/Kg)}{\Delta t\;(s)}\;\times \;3600$$

**Figure 8:**
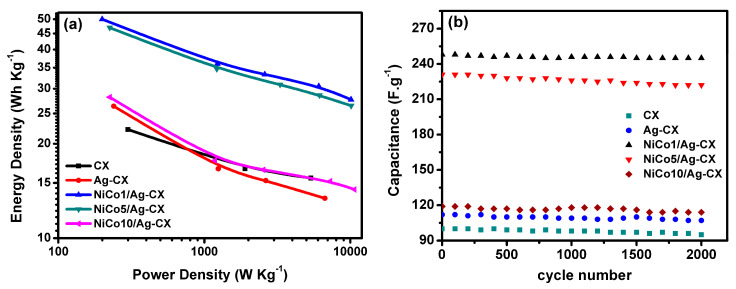
(**a**) Ragone plot, (**b**) stability for all samples at 1A/g over 2000 cycles.

The power densities and energy densities of the assembled symmetric supercapacitors in a 6 M KOH aqueous electrolyte are shown as a Ragone plot in Figure 8a. The NiCo1/Ag-CX and NiCo5/Ag-CX SC devices show very high energy densities of 50 Wh/Kg at a power density of 200 W/Kg and 47 Wh/Kg at a power density of 225 W/Kg, respectively. The higher energy densities of NiCo1/Ag-CX and NiCo5/Ag-CX relative to the others samples are a direct consequence of their low electrode ESRs which in turn provide better accessibility of the ions to the active sites of the electrode [14]. The long-term cycling stability is an important factor in determining whether the SC electrode material will be suitable for real-life practical applications. Figure 8b shows the cycling stability of the symmetric electrodes at current density of 1A·g^−1^ for 2000 cycles. The CX electrodes show an excellent cycling stability with capacitance retention 95% of the initial capacitance after 2000 cycles at a current density of 1 A g^−1^ (see Table 3), and thus, long service electrode lifetimes can be expected [49]. A maximum capacitance retention of about 99% is attained for NiCo1/Ag-CX, which is superior to previously reported values for pure metal oxides and hybrid metal oxides-carbon electrode materials [50,51]. Table 4 presents the specific capacitance, energy density (ED), power density (PD), and capacitance retention (RCs) for NiCo1/Ag-CX, together with reported values for other hybrid NiCo_2_O_4_/carbon electrode materials for comparison. The economic considerations for the prepared composites are very important to facilitate their market use [52]. It is pointed out that our nanocomposites are inexpensive and can be prepared with facile methods in a large scale and their properties can be modified for better electrochemical performance.

## 4. Conclusions

In summary, silver nanoparticles are incorporated into the porous structure of carbon xerogel by the sol-gel method. Silver doped carbon xerogel exhibits a more developed mesopore structure with lower charge transfer resistance, compared to pristine carbon xerogel. Upon functionalization with nickel cobaltite, an increase in the pore diameter of the silver doped carbon xerogel is achieved which enhances the ionic diffusion inside the carbon matrix. The nickel cobaltite functionalized silver doped carbon xerogel electrodes demonstrate highly efficient supercapacitor performance due to their high specific capacitance, low charge transfer resistance and ion diffusion resistance, and excellent stability. Among the different electrode materials presented in the current study, the NiCo1/Ag-CX (Ag-CX functionalized with 1 wt.% of nickel cobaltite) supercapacitor is the optimal choice for electrostatic energy storage due to its small catalytic particle size, uniform particle distribution, open porous structure, and low ESR of 1.9 Ω. Therefore, very small amounts of nickel cobaltite well disperse on the xerogel-Ag matrix can enhance a lot the electro-chemical properties of this composite materials as supercapacitors.

## Figures and Tables

**Table 1:** Textural characteristics of the samples.

Sample	S_BET_	W_0_(N_2_)	L_0_(N_2_)
	m^2^/g	cm^3^/g	nm
CX	462	0.12	4.33
Ag-CX	590	0.24	0.86
NiCo1/Ag-CX	522	0.20	0.89
NiCo5/Ag-CX	811	0.32	1.00
NiCo10/Ag-CX	398	0.17	1.04

**Table 2:** The mean particle size (d_XRD_) of the samples determined from the XRD data.

Sample	d_XRD_ (nm)
Ag-CX	35.2
NiCo1/Ag-CX	26.7
NiCo5/Ag-CX	31.8
NiCo10/Ag-CX	30.9

**Table 3:** Specific capacitances calculated from CV and galvanostatic charge-discharge. Capacitance retention (RCs) and equivalent series resistance (ESR) calculated from EIS.

Samples	CV			Charge-Discharge			ESR	RCs
	C^3mv/s^	C^5mv/s^	C^10mv/s^	C^0.1A/g^	C^0.5A/g^	C^1A/g^		
	F·g^−1^	F·g^−1^	F·g^−1^	F·g^−1^	F·g^−1^	F·g^−1^	Ω	%
CX	119	100	92	160	124	112	16.5	95
Ag-CX	179	172	160	192	122	112	6.8	96
NiCo1/Ag-CX	372	360	342	368	270	248	1.9	99
NiCo5/Ag-CX	360	336	299	341	250	234	3.8	96
NiCo10/Ag-CX	180	179	163	203	128	119	6.4	96

**Table 4:** Electrochemical performance of SCs based on NiCo_2_O_4_/C composite.

Sample	Electrolyte	Configuration	Cs	ED	PD	RCs	Reference
			F·g^−1^	Wh.kg^−1^	KW.kg^−1^	%	
NiCo_2_O_4_/carbon cloth	1M KOH	*Three electrode	245@1A.g^−1^	-	-	95	[53]
CNT/NiCo_2_O_4_	6M KOH	*Three electrode	210@2A.g^−1^	-	-	92	[54]
NiCo_2_O_4_/CA	2M KOH	Two electrode	155@0.5A.g^−1^	47.5	0.4	97	[47]
NiCo_2_O_4_/AC	2M KOH	Two electrode	74@1A.g^−1^	21.4	0.350	95	[27]
AC-NiCo_2_O_4_	6M KOH	Two electrode	54@0.5A.g^−1^	14.7	0.175	85	[55]
carbon aerogel/NiCo_2_O_4_	6M KOH	Two electrode	160@0.5 A.g^−1^	25	1.1085	98	[29]
This work	6M KOH	Two electrode	270@0.5 A.g^−1^	50	0.2	99	-

* Some capacitors have higher capacitance due to different cell configuration.
